# IGF-2-mediated hypoglycemia: a case series and review of the medical therapies for refractory hypoglycemia

**DOI:** 10.1530/EDM-23-0089

**Published:** 2024-03-01

**Authors:** Albert Vu, Constance Chik, Sarah Kwong

**Affiliations:** 1 Division of Endocrinology & Metabolism, Faculty of Medicine & Dentistry, University of Alberta, Edmonton, Alberta, Canada

**Keywords:** Adult, Female, White, Canada, Bone, Liver, Autoimmunity, Growth factors, Tumours and neoplasia, Novel Treatment, March, 2024

## Abstract

**Summary:**

Non-islet cell tumour hypoglycemia (NICTH), typically mediated by insulin-like growth factor 2 (IGF-2), is a rare but highly morbid paraneoplastic syndrome associated with tumours of mesenchymal or epithelial origin. Outside of dextrose administration and dietary modification which provide transient relief of hypoglycemia, resection of the underlying tumour is the only known cure for NICTH. Available medical therapies to manage hypoglycemia include glucocorticoids, recombinant growth hormone, and pasireotide. We report two cases of IGF-2 mediated hypoglycemia. The first was managed surgically to good effect, highlighting the importance of a timely diagnosis to maximise the likelihood of a surgical cure. The second patient had unresectable disease and was managed medically, adding to a growing number of cases supporting the efficacy of glucocorticoids and recombinant growth hormone in NICTH.

**Learning points:**

- Recurrent fasting hypoglycemia in the setting of a malignancy should raise suspicion of non-islet cell tumour hypoglycemia (NICTH), which is typically mediated by IGF-2.
- The initial workup for NICTH should include a serum glucose, C-peptide, insulin, insulin antibodies, beta-hydroxybutyrate, IGF-2, IGF-1, and sulphonylurea screen during a spontaneous or induced hypoglycemic episode.
- An IGF-2/IGF-1 ratio above 10 is highly suggestive of IGF-2-mediated hypoglycemia if the IGF-2 level is normal or elevated. False positives may be seen with sepsis and cachexia as both IGF-2 and IGF-1 are subnormal in these cases. A low IGF binding protein 3 (IGFBP3), such as in renal failure, may also result in a falsely normal or low IGF-2/IGF-1 ratio.
- Surgical resection of the associated tumour is curative in most NICTH cases.
- When the tumour is unresectable, moderate-dose glucocorticoids, low-dose glucocorticoids in combination with recombinant growth hormone, and pasireotide are medical therapies with promising results in controlling NICTH.

## Background

Non-islet cell tumour hypoglycemia (NICTH) is among the rarest forms of hypoglycemia seen in clinical practice. The majority of NICTH cases are mediated by insulin-like growth factor 2 (IGF-2), a bioactive polypeptide that is infrequently overexpressed by tumours of mesenchymal or epithelial origin. IGF-2 exerts insulin-like activity and promotes hypoglycemia by binding to IGF receptors and insulin receptors. Patients with IGF-2-mediated hypoglycemia present with fasting hypoglycemia characterised by suppressed endogenous insulin, ketones, growth hormone, and IGF-1. While the initial management of hypoglycemia is largely supportive, resection of the underlying tumour is curative in most cases (1).

We present two cases of IGF-2-mediated hypoglycemia: the first showing a remarkable response to tumour resection, and the second demonstrating the variable efficacy of different medical therapies in a patient with unresectable disease.

## Case presentation

### Case 1

A healthy 28-year-old woman was found unresponsive during a camping trip with a point-of-care glucose of 1.2 mmol/L. There were no features to suggest sepsis, liver failure, renal failure, heart failure, or adrenal insufficiency. Physical examination was unremarkable.

The patient was resuscitated using intravenous 50% dextrose and transported to a nearby emergency room. She had no known history of diabetes or hypoglycemia either in the fasting or postprandial state. She reported no use of antihyperglycemics or other medications known to cause hypoglycemia.

With recurrent hypoglycemia consistent with Whipple’s triad occurring every 3–4 h despite regular carbohydrate intake, additional investigations were pursued.

### Case 2

A 54-year-old woman with a metastatic hemangiopericytoma was hospitalised with a 2-week history of intermittent confusion, decreased responsiveness, and speech difficulty occurring overnight while fasting. Her past history included a parasagittal hemangiopericytoma treated with surgical resection and radiotherapy 8 years earlier. Six years later, she developed increasing back pain from a metastatic lesion at T5 that required laminectomy. At the time, staging imaging studies showed a solitary lesion in the right lobe of the liver; however, laparotomy found five lesions in all segments of the liver rendering the disease unresectable.

With fasting glucose levels between 1.8 and 2.4 mmol/L, she was managed with intravenous 10% dextrose.

## Investigation

### Case 1

Initial investigations during a hypoglycemic event revealed low serum potassium, C-peptide, insulin, and beta-hydroxybutyrate levels while her creatinine, liver function tests, and complete blood count were normal. Sulphonylureas, insulin analogues, and insulin antibodies were negative. A supervised fast was performed (Table 1). She developed symptomatic hypoglycemia after 3 h, bloodwork was drawn, and she received 1 mg of intravenous glucagon with an increase in serum glucose 10 min later to 4.3 mmol/L and resolution of hypoglycemia symptoms.

**Table 1:** Case 1 fasting chemistries.

	3 h after fast initiation	Reference interval
Glucose (mmol/L)	2.1	3.3–11.0
C-peptide (nmol/L)	<0.02	0.30–1.32
Insulin (pmol/L)	<6.0	35–140
Β-OH (mmol/L)	0.1	<0.4
Serum cortisol (nmol/L)	257	85–620
IGF-1 (μg/L)	45	128–343
IGF-2 (ng/mL)	657	333–967
Sulphonylurea screen	Negative	Negative
Comments	Hypoglycemia symptoms present	

The patient’s IGF-2 was analysed by radioimmunoassay while the IGF-1 was measured using competitive chemiluminescence on the IDS-iSYS analyser. The IGF-2 level was normal but the IGF-1 was suppressed, which yielded an elevated IGF-2/IGF-1 ratio of 14.6.

**Figure 1:**
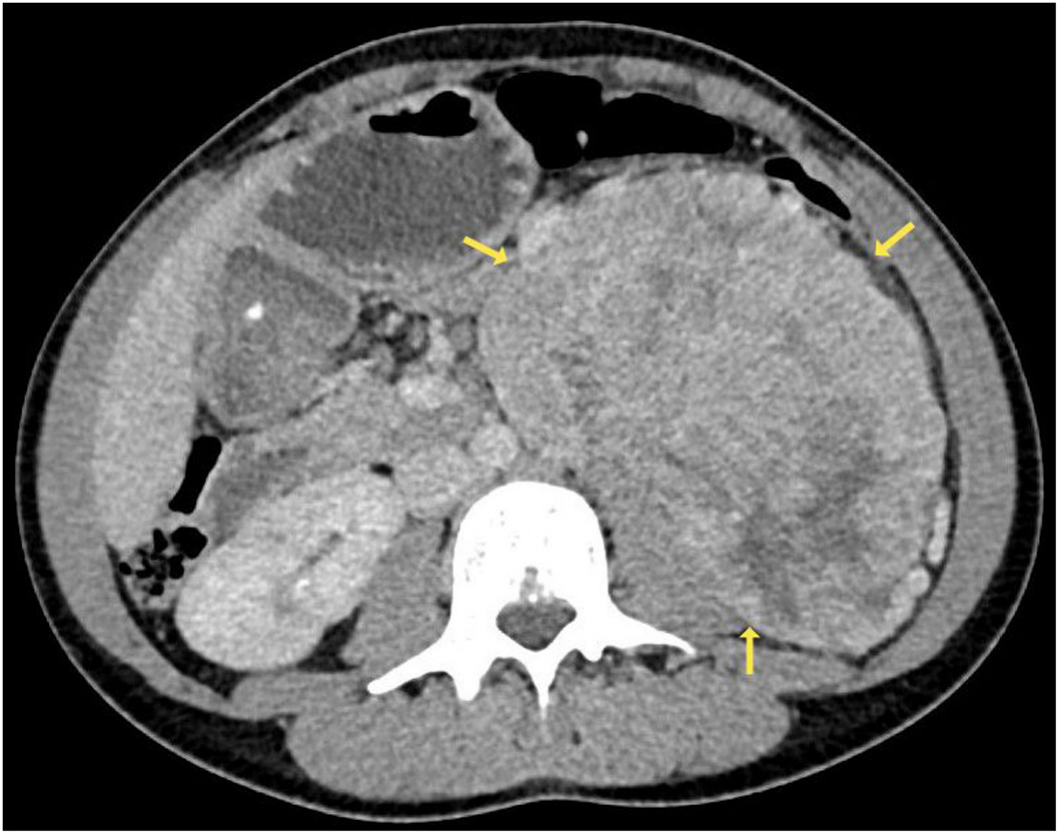
Enhanced CT demonstrating a 13.4 × 9.1 × 15.3 cm lobulated, heterogeneously enhancing retroperitoneal mass with associated left-sided hydronephrosis.

An abdominal ultrasound revealed a 16 × 7.2 × 13 cm left-sided abdominal mass. A contrast-enhanced CT of the abdomen then confirmed the presence of a large, heterogeneously enhancing retroperitoneal mass measuring 15.3 cm × 9.1 × 13.4 cm with associated left-sided hydronephrosis (Fig. 1).

### Case 2

Baseline bloodwork with a glucose of 3.1 mmol/L was significant for low C-peptide, insulin, growth hormone, and IGF-1 levels. A supervised fast was performed with repeat labs done at the time of symptomatic hypoglycemia (Table 2).

**Table 2:** Case 2 baseline and fasting chemistries.

	Baseline	70 min after fast initiation	Reference interval
Glucose (mmol/L)	3.1	2.9	3.3–11.0
C-peptide (nmol/L)	0.14	0.18	0.30–1.32
Insulin (mU/L)	<4.0	9.6	5–20
Growth hormone (μg/L)	<1.0	<1.0	<5.1
Serum cortisol (nmol/L)	478	335	85–620
IGF-1 (μg/L)	39	43	90–360
IGF-2 (μg/L)		951	358–854
IGFBP3 (mg/L)		1.6	2.0–4.0

Hypoglycemia symptoms were present.

In addition to suppressed endogenous insulin production, the patient had an elevated IGF-2 and an elevated IGF-2/IGF-1 ratio of 22.1 during the fast.

She was found to have no evidence of tumour regrowth on MRI brain. Unfortunately, an enhanced CT of the abdomen revealed an increase in the size of her liver metastases.

## Treatment

### Case 1

The patient underwent complete tumour resection and retroperitoneal lymph node dissection. Pathology confirmed a solitary fibrous tumour with negative margins and no metastatic lymphatic involvement. In combination with the elevated IGF-2/IGF-1 ratio, the patient was formally diagnosed with IGF-2-mediated hypoglycemia due to the solitary fibrous tumour, an eponymous condition known as Doege–Potter syndrome (2).

### Case 2

With no indication for surgery, radiation, or chemotherapy, the patient’s nausea and abdominal pain were managed conservatively with antiemetics and analgesics, respectively.

Her hypoglycemia was initially managed with prednisone 30 mg daily and an intravenous dextrose infusion. She suffered increased anxiety from the prednisone which necessitated a dose reduction. Neither 10 mg of prednisone nor 1 mg of dexamethasone was sufficient to control her nocturnal hypoglycemia. She also had no increase in glucose after a subcutaneous dose of octreotide 100 µg. Facing the prospect of a feeding tube, we obtained consent from the patient to initiate a trial of bedtime recombinant growth hormone (rGH) in combination with dexamethasone 1 mg daily to prevent nocturnal hypoglycemia, as pasireotide was not commercially available at the time.

Subcutaneous rGH was started at 0.67 mg daily and gradually increased to 1.67 mg daily, allowing dextrose to be discontinued without hypoglycemia recurrence, with glucose of 5.0 mmol/L and IGF-1 of 159 µg/L.

## Outcome and follow-up

### Case 1

The patient’s hypoglycemia resolved and intravenous dextrose was weaned within a day after surgery. Her IGF-2/IGF-1 ratio reduced to 3.4 after 6 weeks, and she remained euglycemic with no evidence of tumour recurrence 1 year after surgery.

### Case 2

The patient’s hypoglycemia was well controlled with the combination of rGH and low-dose dexamethasone, which allowed her to spend time at home without needing nutritional support via a nasogastric tube until she passed away 6 months later. As she was a patient under palliative care, no follow-up imaging was completed to document any interval change in her liver metastases.

## Discussion

The proposed mechanism of IGF-2-mediated hypoglycemia involves excess tumour production of mature IGF-2 and immature ‘big’ pro-IGF-2, which bind to IGF receptors and insulin receptors (3). This promotes hypoglycemia through various mechanisms including increased peripheral glucose uptake, reduced lipolysis, reduced free fatty acids, reduced hepatic gluconeogenesis, reduced glycogenolysis, and suppressed anterior pituitary growth hormone secretion (4). Patients therefore present with fasting hypoglycemia consistent with Whipple’s triad.

The initial workup typically reveals a suppressed insulin, C-peptide, beta-hydroxybutyrate, IGF-1, growth hormone, and potassium associated with low serum glucose. Measurement of IGF-2 in isolation has limited diagnostic utility as IGF-2 levels are normal in over half of cases (5). In the absence of a commercially available pro-IGF-2 assay, the diagnosis is confirmed by an IGF-2/IGF-1 ratio greater than 10 in the presence of a normal or elevated IGF-2, compared to a normal ratio of 3 or less in healthy subjects (1, 3). A falsely elevated ratio may be seen in patients with sepsis or cachexia due to subnormal IGF-2 and IGF-1 levels in these cases. A low IGF binding protein 3 (IGFBP3), such as in renal failure, may on the other hand cause a falsely normal or low IGF-2/IGF-1 ratio (3). Another limitation of using a fixed cutoff for the IGF-2/IGF-1 ratio is the lack of standardisation between IGF-1 assays, which use different techniques including radioimmunoassay, enzyme-linked immunoassay (ELISA), and competitive chemiluminescence. Even among assays that use the same technique, differences in factors such as calibrators, analytical performance, and sensitivity to IGF binding proteins may result in inter-assay variation in the IGF-1 level (6). As such, we recommend interpreting the IGF-2/IGF-1 ratio with caution and as part of a holistic evaluation of a patient with unexplained fasting hypoglycemia.

Once IGF-2 excess and recurrent hypoglycemia are established, localisation of the underlying tumour is warranted if not already identified. IGF-2-mediated hypoglycemia has been described in a wide variety of epithelial and mesenchymal tumours. Of these, the most commonly implicated are hepatocellular carcinoma, adrenocortical carcinoma, mesothelioma, hemangiopericytoma, and solitary fibrous tumours (3).

The first-line treatments for hypoglycemia are oral and parenteral dextrose administration. Contrary to other pathologies, hypoglycemia mediated by IGF-2 often recurs despite best efforts to optimise carbohydrate and protein intake. When there is localised disease, surgical resection is curative with resolution of hypoglycemia and normalisation of the IGF-2/IGF-1 ratio in most cases. In patients with metastatic or otherwise incurable disease, tumour debulking can be considered to alleviate hypoglycemia. Systemic chemotherapy, radiotherapy, and arterial embolisation have also successfully eliminated hypoglycemia in select cases of unresectable tumours (7, 8).

Several pharmacologic agents have shown efficacy in NICTH. Glucocorticoids mitigate hypoglycemia by suppressing pro-IGF-2 production, increasing hepatic gluconeogenesis, promoting lipolysis, and reducing peripheral glucose uptake (3). Prednisone, prednisolone, methylprednisolone, and dexamethasone are readily available and control hypoglycemia in reversible fashion when doses exceed a patient-specific threshold. The minimum effective dose in one review of NICTH with solitary fibrous tumours was 25 mg/day for prednisolone, 32 mg/day for methylprednisolone, and 2 mg/day for dexamethasone (2). As in our second case, their efficacy may be limited by dose-dependent adverse effects. Recurrent hypoglycemia is also seen when the glucocorticoid dose is reduced below the aforementioned thresholds, limiting their long-term utility (1, 2, 9).

Monotherapy with high-dose subcutaneous recombinant growth hormone (rGH) is effective in NICTH, albeit limited by adverse effects such as pitting oedema, skin tags, and exacerbation of arthritic pain (10). The combination of rGH with glucocorticoids at low doses, as in case 2, is an effective regimen with minimal adverse effects in patients with inoperable tumours (2, 11) even though the use of rGH is typically contraindicated in patients with an advanced tumour.

Diazoxide, which can mitigate NICTH in the short term, has had inconsistent results when used alone or in combination with other agents (1, 12).

Somatostatin analogues may reduce NICTH by binding to somatostatin receptors (SSTRs) which inhibit insulin and glucagon secretion. Octreotide, a first-generation somatostatin analogue, has been largely ineffective even in high doses or as a continuous infusion. On the other hand, the second-generation somatostatin analogue pasireotide is well known to cause hyperglycemia and has been used with marked improvement in one NICTH case. The efficacy of pasireotide is attributed to its higher binding affinity for SSTRs, leading to reduced insulin secretion, reduced incretin secretion, and a greater reduction in pro-IGF-2 titres compared to octreotide (12). Indeed, pasireotide would have been tried in case #2 had it been commercially available at the time.

## Conclusion

IGF-2-mediated hypoglycemia is an exceedingly rare and probably under-recognised cause of hypoglycemia in the setting of epithelial or mesenchymal tumours. While surgical resection is curative in most cases, refractory hypoglycemia due to unresectable tumours remains a challenge. Of the available medical therapies for NICTH, in patients with suboptimal response or intolerance to moderate-dose glucocorticoids, low-dose glucocorticoids in combination with rGH, and pasireotide can be considered.

## Declaration of interest

The authors declare that there is no conflict of interest that could be perceived as prejudicing the impartiality of the study reported.

## Funding

This research did not receive any specific grant from any funding agency in the public, commercial, or not-for-profit sector.

## Patient consent

Every effort was made to contact the patient in case 1 and next of kin for the patient in case 2 to obtain consent but was unsuccessful.

## Author contribution statement

AV wrote the manuscript. CC was the physician responsible for the patient in case 2 and contributed to the editing of the manuscript. SK was the physician responsible for the patient in case 1 and contributed to the editing of the manuscript.
